# The social welfare effect of e-commerce product reputation information asymmetry from the perspective of network externality

**DOI:** 10.1371/journal.pone.0313852

**Published:** 2025-01-02

**Authors:** Min Zhao, Shuyun Wang, Tongshui Xia

**Affiliations:** 1 Business School, Shandong Normal University, Jinan, China; 2 Business School, Shandong Management University, Jinan, China; 3 School of Economics and Management, Yantai University, Yantai, China; Zhejiang Gongshang University, CHINA

## Abstract

Reputation is the most important intangible asset of merchants. In the e-commerce platform market, reputation information has become an important signal of product quality. However, with increasingly fierce competition among merchants on these platforms, violations of reputation information, such as “click farming,” “cash rebate for favorable comments,” and “pay per click,” have caused information asymmetry and adverse selection. Based on the network externality perspective, considering the duopoly e-commerce platform market, this paper uses game theory to construct a theoretical model to compare and analyze the changes in consumers, merchants, platforms, and social total welfare when the reputation information of e-commerce products is symmetric and asymmetric. The research results show that when the reputation information of e-commerce products is symmetrical, the reputation mechanism of the e-commerce platform can play a positive role, the platform income decreases, and the consumer surplus and the total social welfare level increase. The increment increases with the increase in consumer-side network externality, and the e-commerce platform transfers part of the surplus value to consumers. Due to the influence of network externality, reputation information asymmetry, and violation penalty cost, reputation asymmetry decreases consumer, merchant, and total social welfare and increases platform profits, indicating that the e-commerce platform lacks the economic motivation to govern the violation of reputation information. We recommend that the healthy development of e-commerce platforms proceeds from three aspects: building a reputation mechanism for e-commerce platforms that is jointly supervised by e-commerce platforms, third-party institutions, and social organizations; increasing the cost of punishment for violations; and exerting platform network effects to enhance the competitiveness of enterprises.

## Introduction

In recent years, e-commerce has developed rapidly, but the phenomenon of poor-quality goods “squeezing out” quality goods is frequent, and the e-commerce market has even been labeled a “lemon market” [1]. Online reviews, sales rankings, and other reputation information are important factors for consumers to judge the quality of e-commerce products and make consumption decisions. When information asymmetry occurs, merchants with sufficient information are in an advantageous position, while consumers with poor information are at a disadvantage, leading to adverse selection [2]. Not only does this waste consumers’ time and money, but, more importantly, it distorts the marketplace and undermines the foundations of honest competition.

The reputation information of e-commerce products mainly includes consumer feedback evaluation, sales rankings, credit ratings, and real name certification [3], among which feedback evaluation and sales rankings play a significant role in the reputation mechanism [4]. False reputation information, such as “click farming,” “cash rebates for favorable comments,” and “pay per click,” has a low cost and strong operability, and has become a “heavy disaster area” with frequently asymmetric reputation information. Therefore, we focus our research on reputation information such as feedback evaluation and sales ranking.

To improve the “lemon market” phenomenon, some efforts have been made. During the period from October 13, 2020 to May 17, 2021, Sam’s Club APP was fined 300,000 yuan for violating the Anti-Unfair Competition Law due to its default five-star positive reviews (https://finance.sina.com.cn). Amazon has long strictly prohibited false information and other behaviors on its platform. In 2023, the company used machine learning and artificial intelligence (AI) technologies and professional expert investigation teams to monitor and block false reviews. It proactively blocked more than 250 million suspected false reviews on its platform, and took legal action against more than 150 bad actors involved in review abuse in the United States, Europe, and China (https://www.takungpao.com). However, review manipulation is persistent, and the rise of generative AI has made it easier than ever for bad actors to write fake reviews. In August 2024, the U.S. Federal Trade Commission (FTC) issued a ban on fake e-commerce reviews that explicitly prohibits companies from knowingly buying, selling, or promoting fake online reviews, including AI reviews, and all forms of fake review behavior are regulated in detail (https://www.163.com). It can be seen that the problem of asymmetric information about e-commerce product reputation is receiving increasing attention.

Network externalities are the basic attributes of the e-commerce market, which means that the value of connecting to a network depends on the number of others connected to the network [5]. In general, the more users there are, the higher the utility of each user. Network externalities are divided into direct and indirect network externalities. Direct network externalities refer to interactions between users on the same side (i.e., the same type of users, whether buyers or sellers) through platform interaction; indirect network externalities refer to interactions between users on both sides of the platform, such as buyers and sellers [6]. The network externality of the e-commerce market is increasingly affected by reputation information. Real reviews increase user value and promote the growth of network scale, while review manipulation has a negative impact [7]. Scholars have also found that network externalities can affect the social welfare effect by influencing the peer effect and conformity behavior of consumers [8]. Therefore, we aimed to explore the theoretical mechanism of the impact of e-commerce product reputation information asymmetry on social welfare from the perspective of network externalities to provide a theoretical basis for the governance of e-commerce platform reputation information asymmetry.

We sought to answer the following questions: (1) Can the e-commerce product reputation mechanism effectively increase the total welfare of consumers, merchants, platforms, and society? (2) How do consumers, businesses, platforms, and total social welfare change when the information is asymmetric? (3) What are the factors that affect these changes? To answer the above questions, in this study, which we based on the perspective of network externalities, considering the background of China’s duopolistic e-commerce platform based on the Hotelling model, we took consumer online feedback evaluation and sales ranking as the main research object of e-commerce product reputation information asymmetry, and discussed the changes in consumer utility, merchant utility, platform revenue, and total social welfare when e-commerce product reputation information is asymmetric.

Our research contributions are reflected in the following two aspects. First, we theoretically demonstrate the impact of e-commerce product reputation information asymmetry on platform stakeholders and social welfare. Making up for the lack of uniformity of empirical conclusions, this enriches theory on the e-commerce reputation mechanism and provides a theoretical reference for future empirical research. Second, it discusses the factors influencing social welfare in the e-commerce market and provides some ideas and references for the role of reputation mechanisms in the e-commerce market and the formulation of platform governance strategies.

## Literature review

Scholars have previously studied network externalities, e-commerce platform reputation mechanisms, and e-commerce product reputation information asymmetry and its impact, laying a solid foundation for the development of this study.

### Network externalities

Previous research by experts and scholars on network externalities focuses mainly on their strategic role in platform competition and platform value as well as pricing strategy. Cusumano et al. analyzed multilateral market platforms, such as Apple, Google, and Microsoft, through a case comparison, and found that platforms can create and enhance direct and indirect network externalities and promote platform competitiveness by regulating key elements [9]. Zhu and Liu’s empirical research revealed that the direct and indirect network effects of multilateral market platforms significantly increased platform sales and revenue [10]. Liano et al. found through pricing game modeling that platforms’ low-price strategies can strengthen network externalities, attract more users, increase competitors’ user transfer costs, and maintain platforms’ competitive advantages [11]. Zhang et al. investigated cross-network externalities, constructed a recovery pricing model, and studied the investment and pricing strategies of value-added services of multilateral distribution platforms [12]. Li and Gao also studied the impact of network externalities on online medical platforms [13] and Waste Electrical and Electronic Equipment recycling platforms [14], and proposed corresponding product pricing strategies. From the literature, we can see that existing studies have investigated the role of network externalities on different types of platforms through case studies, empirical evidence, and theoretical modeling, but none have considered the problem of network externalities affected by reputation information asymmetry in bilateral markets.

### E-commerce platform reputation mechanisms

Experts and scholars have long studied reputation mechanisms and their effectiveness. After the KMRW reputation model was proposed, Shapiro was the first to find that reputation premiums can motivate firms to improve the quality of their products and services and abandon the speculative behavior of lowering quality to gain short-term benefits [15]. Since then, scholars such as Resnick et al., Melnik and Alm, and Houser and Wooders have used data from online auctions on eBay to empirically demonstrate the effect of product reputation on price, sales, and quality [16–18]. Qian and Zhang used data from Chinese e-commerce platforms to argue that reputation mechanisms can effectively mitigate the adverse selection problem [19, 20].

With the continuous emergence of reputation “noise” [19], scholars have launched a heated discussion on the effectiveness of reputation mechanisms. Resnick and Zeckhauser found that most eBay consumers do not participate in reviews, while those who actively review tend to choose positive reviews [21]. Jin and Kato argued that eBay’s ranking mechanism and anonymity allow speculative sellers to obtain top rankings at low cost and to “restore reputation” by changing accounts after selling low-quality goods that damage their reputation, leading to the failure of the binding force of reputation [22]. Scholars found that if only consumer evaluation is used as the main content of the reputation mechanism, the reputation signal cannot significantly affect product sales or encourage merchants to improve product quality [23], quality certification can be used as a supplement [24]. In addition, consumers’ attitudes towards false marketing and promotion of e-commerce products were more negative than their attitudes towards actual sales fraud, which may cause more damage to the reputation of platform-based e-commerce [4]. From these studies, it is not difficult to see that the effectiveness of the reputation mechanism is closely related to the asymmetry of reputation information in the two-sided market of e-commerce platforms.

### E-commerce product reputation information asymmetry and its impact

Academic research on demand information asymmetry and cost information asymmetry is extensive, but research on e-commerce product reputation information asymmetry is lacking. The reputation information asymmetry of e-commerce platforms that we propose refers to the inconsistency between the “expected product reputation information” seen by consumers and the “real product reputation information,” which is caused by violations such as the manipulation of comments. The expected reputation tends to be greater than the real reputation, essentially reflecting an asymmetry in quality information.

Akerlof suggested that adverse selection caused by quality information asymmetry is the root cause of market failure [25]. Zhou et al. found that in product-differentiated markets, when product quality information is asymmetric, monopolistic firms have an incentive to use false quality [26], and whether a firm uses false quality depends on the additional marketing cost and the penalty cost of being discovered after using false information [27]. Wang defined the concept of the degree of quality information asymmetry and discussed the impact of changes in quality information asymmetry on consumer utility and firm profit [28]. The abovementioned studies provide the research basis for the model construction described in this paper.

Research on the impact of asymmetric information of e-commerce product reputation focuses on consumer decision-making, product sales, and platform revenue and is mainly carried out using empirical methods. In terms of consumer decision-making, through scenario experiments, Liu and Wang found that review manipulation leads to a significant decrease in consumers’ perceptions of the usefulness and trustworthiness of online reviews, and purchase intention decreases significantly [29, 30]. However, Zhong demonstrated empirically, using questionnaires and commercial data, that fake online reviews were positively related to consumer purchase decisions [31]. In terms of product sales, some have shown that fake reviews have a negative impact on product sales [32], others have proposed an inverted U-shaped relationship, i.e. that the small-scale use of fake reviews would boost product sales, but once a critical value is exceeded, it would inhibit performance [33, 34]. Chen found that false reviews lead to increased transaction costs for consumers and merchants, and the platform suffers as a result. However, if merchants choose to improve reputation ratings by manipulating reviews, consumers perceive higher-quality goods in the short term, and the platform benefits as a result. Nevertheless, as consumers’ purchasing experience on the platform increases, the higher reputation ratings caused by false reviews fail to convey high-quality signals, consumer perceived goods quality decreases, and therefore platform gains are impaired [35]. Zhang found that the quantity of review information has a positive impact on social welfare, but quality information and matching information play different roles in the welfare enhancement process, and a higher manipulation cost factor can alleviate the prisoner’s dilemma of sellers and increase consumer welfare [36].

The above studies researched the welfare utility of e-commerce market network externality and reputation information asymmetry to consumers, merchants, and platforms from different perspectives but did not comprehensively consider their interaction relationships. The conclusions of empirical research are not uniform, which is related to the difficulty of obtaining fake review data, the accuracy of manual labeling, and the applicability of research methods. Therefore, this paper avoids the empirical approach and explores the theoretical mechanism of the impact of e-commerce product reputation information asymmetry on platform stakeholders and social welfare from the perspective of network externalities.

## Modeling

The Hotelling model is a classic model of spatial competition. It mainly analyzes how firms compete in a limited market space. The two-sided market and the network effect of the e-commerce platform increase the complexity of the competition. Therefore, we extended the Hotelling model to better fit our research scenario. Referring to the model settings of Armstrong and Wright [37], Zhou [38], Yu [7], and Xie [39], and the characteristics of China’s duopolistic e-commerce platforms Tmall and Jingdong, and assuming that different e-commerce platforms have differences for both merchants and consumers, we constructed the consumer, merchant and platform profit utility models. Different from their models, we refer to the definition of quality information asymmetry by Wang [28] and incorporate quality information asymmetry into the models.

### Model assumptions

We form a linear city of length 1, in which the e-commerce platform T is located at the left end of the city and the e-commerce platform J is located at the right end of the city, and the e-commerce platform has two types of users, consumers (b) and merchants (s), uniformly distributed along a line with a total number of 1. The user’s location represents their ideal choice of platform. Since there are two choices in the market, each user incurs a certain transportation cost, denoted by t_b_ and t_s_ for buyers and sellers, respectively. E-commerce platform transportation costs reflect platform differences, but also represent the choice of user preferences. Users joining the platform will receive a basic utility (θ), which is assumed to be large enough to allow the duopoly to cover all users in the market. Individual buyers or sellers join the platform by paying a certain amount of p or w.

Merchant participation in the market generates an indirect network externality with a coefficient β_s_ (0 < α_b_ < 1), and consumer participation in the market generates both an indirect network externality with a coefficient β_b_ (0 < α_b_ < 1) and a direct network externality with a coefficient α_b_ (0 < α_b_ < 1). Indirect network externality exists on both sides because an increase in user size on both sides attracts users on the other side, and both have positive utility. The direct network externality on the merchant side has both learning utility (positive utility) and competitive utility (negative utility) and was assumed to be zero here to simplify the model calculation. The direct network externality on the consumer side mainly arises from the feedback evaluation of different consumers and sales rankings to help consumers’ decision-making and is of positive utility. Therefore, in this paper, the consumer-side direct network externality is used to characterize the e-commerce product reputation information, and if there is information asymmetry, the direct network externality is regulated by the degree of reputation information asymmetry $\left.\begin{array}{c} i=\left.\left|\frac{m_{0}-m_{y}}{m_{0}}\right|\right. \end{array}\right.$ [22], where m_0_ is the real product reputation information of the e-commerce product and m_y_ is the expected product reputation information of the consumer. According to reality, when false reputation information exists, the expected product reputation information of the consumer is often larger than the real reputation information of the product; that is m_y_ > m_0_, so $i=\frac{m_{y}-m_{0}}{m_{0}}$, assuming 0 ≤ i ≤ 1.

**Fig 1:**
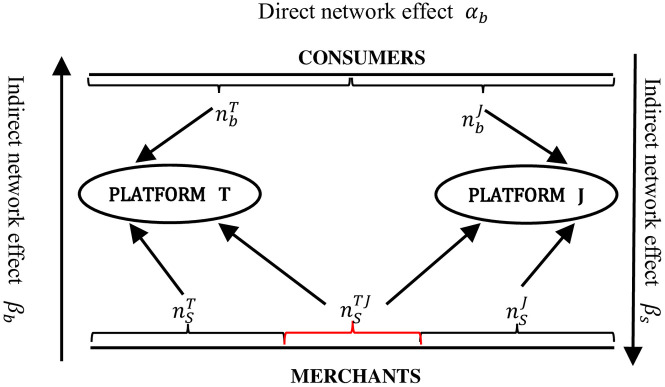
Market structure of e-commerce platforms.

In addition, assuming that merchants can choose single-homing or multi-homing and consumers choose single-homing, the number of single-homing and multi-homing merchants on platforms T and J is denoted by $n_{s}^{T}\;n_{s}^{J},$ and $n_{s}^{T,J}$, respectively, and the number of single-homing consumers is denoted by $n_{b}^{T}$ and $n_{b}^{J}$, respectively. According to the previous assumptions, it is known that $n_{s}^{T\;}+n_{s\;}^{J}+n_{s}^{T,J}=1$, $n_{b}^{T}+n_{b}^{J}=1$. Fig 1 shows the market share structure of e-commerce platforms. The consumer and merchant utilities of platform T are denoted by $U_{b}^{T}$ and $U_{s}^{T}$, the consumer and merchant utilities of platform J are denoted by $U_{b}^{J}$ and $U_{s}^{J}$, the profits obtained by the platform are π^T^ and π^J^, and the platform merchant reputation information violation generates a penalty cost of f. The model parameters and variables are defined in Table 1.

**Table 1:** Model parameters and variables.

Parameter	Definition	Parameter	Definition
**θ**	Base utility	**α**_**b**_	Direct network utility coefficient on the consumer side
**β**_**s**_	Indirect network utility coefficient on the merchant side	**β**_**b**_	Indirect network utility coefficient on the consumer side
**w** ^**T**^	Merchant pricing on Platform T	**p** ^**T**^	Consumer pricing on Platform T
**w** ^**J**^	Merchant pricing on Platform J	**p** ^**J**^	Consumer pricing on Platform J
**t**_**b**_	Transportation cost on the consumer side	**t**_**s**_	Transportation cost on the merchant side
$n_{s}^{T}$	Number of merchants single-homing on platform T	$n_{s}^{J}$	Number of merchants single-homing on platform J
$n_{s}^{T,J}$	Number of merchants multi-homing on platforms T and J	$n_{b}^{T}$	Number of consumers single-homing on platform T
$n_{b}^{J}$	Number of consumers single-homing on platform J	$U_{b}^{T}$	Utility of consumers single-homing on platform T
$U_{s}^{T}$	Utility of merchants single- homing on platform T	$U_{b}^{J}$	Utility of consumers single-homing on platform J
$U_{s}^{J}$	Utility of merchants single-homing on platform J	$U_{s}^{T,J}$	Utility of merchants multi-homing on platforms T and J
**Π** ^**T**^	Platform T profit	**Π** ^**J**^	Platform J profit
**i**	Degree of reputation information asymmetry	**m**_**0**_	Real product reputation information
**m**_**y**_	Expected product reputation information	**f**	Penalty cost of reputation information violation

### Basic model

The game between the duopoly platform and the bilateral users consists of two phases. In the first phase, platform T and platform J set pricing strategies (p^T^, w^T^) and (p^J^, w^J^) simultaneously, while in the second phase, the bilateral users observe the pricing and make their own participation decisions, and both platforms determine the market size. We used the inverse induction method to solve this dynamic game. The basic model of this paper is consumer utility, business utility, platform profit, and total social welfare when an e-commerce platform has no reputation mechanism, which means that the direct network externality on the consumer side is zero. At this time, consumers rely completely on product promotion information to make independent decisions on whether to consume. There is no direct network externality at the consumer’s end. The utility of consumers located on the T platform is

$$U_{b}^{T}=\theta +\beta_{b}{(n}_{s}^{T}+n_{s}^{T,J})-p^{T}-t_{b}n_{b}^{T}.$$ (1)

Similarly, the utility of a consumer located on platform J is

$$U_{b}^{J}=\theta +\beta_{b}(n_{s}^{J}+n_{s}^{T,J})-p^{J}-t_{b}{(1-n}_{b}^{T}).$$ (2)

The utility of merchants single-homing to platform T is

$$U_{s}^{T}=\theta +\beta_{s}n_{b}^{T}-w^{T}-t_{s}n_{s}^{T}.$$ (3)

The utility of merchants single-homing to platform J is

$$U_{s}^{J}=\theta +\beta_{s}n_{b}^{J}-w^{J}-t_{s}{(1-n}_{s}^{T}-n_{s}^{T,J}).$$ (4)

The utility of merchants multi-homing to platforms T and J is

$$U_{s}^{T,J}=\theta +\beta_{s}\left(n_{b}^{T}+n_{b}^{J}\right)-w^{T}-w^{J}-t_{s}.$$ (5)

Let (1) = (2), (3) = (5), and (4) = (5) to obtain the undifferentiated position:

$$n_{b}^{T}=\frac{1}{2}-\frac{t_{s}\left(p^{T}-p^{J}\right)+\beta_{b}(w^{T}-w^{J})}{2\left.\left(t_{b}-\beta_{b}\beta_{s}\right)\right.}$$

$$n_{s}^{T}=\frac{2t_{s}-\beta_{s}+2w^{J}}{2t_{s}}-\frac{{\beta_{s}t}_{s}\left(p^{T}-p^{J}\right)+\beta_{b}\beta_{s}\left(w^{T}-w^{J}\right)}{2t_{s}\left.\left(t_{b}-\beta_{b}\beta_{s}\right)\right.}$$

$$n_{s}^{T,J}=\frac{\beta_{s}-t_{s}-(w^{T}+w^{J})}{t_{s}}.$$ (6)

According to $n_{s}^{T}+n_{s\;}^{J}+n_{s}^{T,J\;}=1$, $n_{b}^{T}+n_{b}^{J}=1$, it can be found that

$$n_{b}^{J}=\frac{t_{s}\left(p^{T}-p^{J}\right)+\beta_{b}(w^{T}-w^{J})}{2\left.\left(t_{b}-\beta_{b}\beta_{s}\right)\right.}-\frac{1}{2}$$

$$n_{s}^{J}=1-\frac{2t_{s}+\beta_{s}-2w^{T}}{2t_{s}}+\frac{{\beta_{s}t}_{s}\left(p^{T}-p^{J}\right)+\beta_{b}\beta_{s}\left(w^{T}-w^{J}\right)}{2t_{s}\left.\left(t_{b}-\beta_{b}\beta_{s}\right)\right.}$$ (7)

At this point, the profits of platforms T and J are:

$$\pi^{T}=p^{T}n_{b}^{T}+w^{T}(n_{s}^{T}+n_{s}^{T,J})$$

$$\pi^{J}=p^{J}n_{b}^{J}+w^{J}(n_{s}^{J}+n_{s}^{T,J})$$ (8)

Taking (6) and (7) into (8) and taking the first-order derivatives of p^T^, p^J^, w^T^, and w^J^, we obtain the bilateral pricing in equilibrium as

$$p^{T}=p^{J}=t_{b}-\beta_{s}\frac{3\beta_{b}+\beta_{s}}{4t_{s}}$$

$$w^{T}=w^{J}=\frac{\beta_{s}\text{-}\beta_{b}}{4}.$$ (9)

Substituting (9) into (6), (7), and (8) yields the market share of the bilateral users of the platform under equilibrium:

$$n_{b}^{T}=n_{b}^{J}=\frac{1}{2},n_{s}^{T}=\frac{4t_{s}-\beta_{b}-\beta_{s}}{4t_{s}},n_{s}^{T,J}=\frac{\beta_{b}+{\beta_{s}-2t}_{s}}{2t_{s}},n_{s}^{J}=\frac{4t_{s}-\beta_{b}-\beta_{s}}{4t_{s}}.$$

The platform profits are $\pi^{T}=\pi^{J}=\frac{1}{2}t_{b}-\frac{\beta_{s}^{2}+6\beta_{s}\beta_{b+}\beta_{b}^{2}}{16t_{s}}$.

In equilibrium, all consumer surplus (CS) is

$$\int_{0}^{\frac{1}{2}}\left[\theta +\beta_{b}{(n}_{s}^{T}+n_{s}^{T,J})-p^{T}-t_{b}n_{b}^{T}\right]d_{t_{b}}+\int_{0}^{\frac{1}{2}}\left[\theta +\beta_{b}(n_{s}^{J}+n_{s}^{T,J})-p^{J}-t_{b}{(1-n}_{b}^{T})\right]d_{t_{b}}=\theta -\frac{3}{8}+\frac{\beta_{s}^{2}+4\beta_{s}\beta_{b+}\beta_{b}^{2}}{4t_{s}}$$ (10)

In equilibrium, all merchant surplus (PS) is

$$\int_{0}^{\frac{4t_{s}-\beta_{b}-\beta_{s}}{4t_{s}}}\left[\theta +\beta_{s}n_{b}^{T}-w^{T}-t_{s}n_{s}^{T}\right]d_{t_{s}}+\int_{0}^{\frac{4t_{s}-\beta_{b}-\beta_{s}}{4t_{s}}}\left[\theta +\beta_{s}n_{b}^{J}-w^{J}-t_{s}{(1-n}_{s}^{T}-n_{s}^{T,J})\right]d_{t_{s}}+\int_{0}^{\frac{\beta_{b}+{\beta_{s}-2t}_{s}}{2t_{s}}}\left[\theta +\beta_{s}\left(n_{b}^{T}+n_{b}^{J}\right)-w^{T}-w^{J}-t_{s}\right]d_{t_{s}}=\theta +\frac{1}{2}\beta_{s}+\frac{1}{2}\beta_{b}-\frac{{\left(4t_{s}-\beta_{b}-\beta_{s}\right)}^{2}+{\left(\beta_{b}+\beta_{s}-2t_{s}\right)}^{2}}{16t_{s}^{2}}$$ (11)

In equilibrium, the profits of the two platforms are

$$\pi^{T}+\pi^{J}=-\frac{\beta_{s}^{2}+6\beta_{s}\beta_{b+}\beta_{b}^{2}}{8t_{s}}$$ (12)

From Eqs (10), (11), and (12), the total level of social welfare of the platform system in the absence of the reputation mechanism can be obtained as

$$\begin{array}{ll} {\text{W}}_{1} & =\text{CS}+\text{PS}+\pi^{\text{T}}+\pi^{\text{J}}\\ \; & =2\theta -\frac{3}{8}+\frac{1}{2}\beta_{\text{s}}+\frac{1}{2}\beta_{\text{b}}+{\text{t}}_{\text{b}}+\frac{{\left(\beta_{\text{b}}+\beta_{\text{s}}\right)}^{2}}{8{\text{t}}_{\text{s}}}-\frac{{\left(4{\text{t}}_{\text{s}}-\beta_{\text{b}}-\beta_{\text{s}}\right)}^{2}+{\left(\beta_{\text{b}}+\beta_{\text{s}}-2{\text{t}}_{\text{s}}\right)}^{2}}{16{\text{t}}_{\text{s}}^{2}} \end{array}$$ (13)

Eqs 10–13 show the consumer surplus, merchant surplus, platform profit, and total level of social welfare in the e-commerce market in equilibrium in the basic model; that is, when there is no reputation mechanism.

### Total social welfare levels when e-commerce product reputation information is symmetric

When the e-commerce platform is designed with a reputation mechanism, consumers provide real evaluation feedback on the products they purchase, and the platform system recommends products for search users based on sales data. Merchants and consumers are fully informed and consistent on reputation information, which increases the direct network externalities on the consumers’ side. The model of consumer utility, merchant utility, and platform profit is as follows:

The utility of consumers located on platform T is

$$U_{b}^{T^{'}}=\theta +\alpha_{b}n_{b}^{T}+\beta_{b}{(n}_{s}^{T}+n_{s}^{T,J})-p^{T}-t_{b}n_{b}^{T}.$$ (14)

Similarly, the utility of a consumer located on platform J is

$${U_{b}^{J}}^{'}=\theta +\alpha_{b}n_{b}^{J}+\beta_{b}(n_{s}^{J}+n_{s}^{T,J})-p^{J}-t_{b}{(1-n}_{b}^{T}).$$ (15)

The merchant utility and platform profitability remain unchanged:

$${U_{s}^{T}}^{'}=\theta +\beta_{s}n_{b}^{T}-w^{T}-t_{s}n_{s}^{T}$$

$${U_{s}^{J}}^{'}=\theta +\beta_{s}n_{b}^{J}-w^{J}-t_{s}{(1-n}_{s}^{T}-n_{s}^{T,J})$$ (16)

$$U_{s}^{T,J^{'}}=\theta +\beta_{s}\left(n_{b}^{T}+n_{b}^{J}\right)-w^{T}-w^{J}-t_{s}$$

$${\pi^{T}}^{'}={\pi^{J}}^{'}=p^{J}n_{b}^{J}+w^{J}(n_{s}^{J}+n_{s}^{T,J}).$$

Similar to the previous calculation process, under equilibrium, consumer pricing, merchant pricing, the platform user market share, consumer surplus, merchant surplus, platform profit, and platform system social welfare are as follows:

$$p^{T^{‘}}=p^{J^{’}}=t_{b}-\alpha_{b}-\beta_{s}\frac{3\beta_{b}+\beta_{s}}{4t_{s}}.$$ (17)

$$w^{T^{‘}}=w^{J^{’}}=\frac{\beta_{s}-\beta_{b}}{4}.$$ (18)

$$n_{b}^{T^{‘}}=n_{b}^{J‘}=\frac{1}{2},n_{s}^{T^{‘}}=\frac{4t_{s}-\beta_{b}-\beta_{s}}{4t_{s}},n_{s}^{T,J‘}=\frac{\beta_{b}+{\beta_{s}-2t}_{s}}{2t_{s}},n_{s}^{J‘}=\frac{4t_{s}-\beta_{b}-\beta_{s}}{4t_{s}}.$$ (19)

$$CS'=\theta -\frac{3}{8}+\frac{3}{2}\alpha_{b}+\frac{\beta_{s}^{2}+4\beta_{s}\beta_{b+}\beta_{b}^{2}}{4t_{s}}.$$ (20)

The merchant surplus is

$$PS'=\theta +\frac{1}{2}\beta_{s}+\frac{1}{2}\beta_{b}-\frac{{\left(4t_{s}-\beta_{b}-\beta_{s}\right)}^{2}+{\left(\beta_{b}+\beta_{s}-2t_{s}\right)}^{2}}{16t_{s}^{2}}.$$ (21)

The profits of the two platforms are

$$\pi^{T‘}+\pi^{J‘}=t_{b}-\alpha_{b}-\frac{\beta_{s}^{2}+6\beta_{s}\beta_{b+}\beta_{b}^{2}}{8t_{s}}.$$ (22)

The total social welfare level of the platform system is

$$\begin{array}{ll} {\text{W}}_{2} & =\text{CS'}+\text{PS'}+\pi^{\text{T‘}}+\pi^{\text{J‘}}\\ \; & =2\theta -\frac{3}{8}+\frac{1}{2}\alpha_{\text{b}}+\frac{1}{2}\beta_{\text{s}}+\frac{1}{2}\beta_{\text{b}}+{\text{t}}_{\text{b}}+\frac{{\left(\beta_{\text{b}}+\beta_{\text{s}}\right)}^{2}}{8{\text{t}}_{\text{s}}}-\frac{{\left(4{\text{t}}_{\text{s}}-\beta_{\text{b}}-\beta_{\text{s}}\right)}^{2}+{\left(\beta_{\text{b}}+\beta_{\text{s}}-2{\text{t}}_{\text{s}}\right)}^{2}}{16{\text{t}}_{\text{s}}^{2}}. \end{array}$$ (23)

Eqs. (20)–(23) show the consumer surplus, merchant surplus, platform profit, and total social welfare levels in the e-commerce market in equilibrium when there is a reputation mechanism and the information is symmetric. Does the reputation mechanism play a positive role? We compared the results of the calculations in this section with the basic model and obtained

$${\Delta p}^{T}={\Delta p}^{J}=p^{T^{‘}}-p^{T}=p^{J^{‘}}-p^{J}=-\alpha_{b}<0$$

$${\Delta w}^{T}={\Delta w}^{J}=w^{T^{‘}}-w^{T}=w^{J^{‘}}-w^{J}=0$$

$$\Delta n_{b}^{T}=0,\Delta n_{b}^{J}=0,\Delta n_{s}^{T}=0,{\Delta n}_{s}^{T,J}=0,\Delta n_{s}^{J}=0$$

$$\Delta CS=CS'-CS=\frac{3}{2}\alpha_{b}>0$$

ΔPS=PS'-PS=0

$$\Delta \pi =\left(\pi^{T‘}+\pi^{J‘}\right)-\left(\pi^{T}+\pi^{J}\right)=-\alpha_{b}<0$$

$$W_{2}\text{-}W_{1}=\frac{1}{2}\alpha_{b}>0.$$ (24)

It can be seen that under the reputation mechanism and when information is symmetric, the platform has lowered pricing for consumers, and consumer welfare has risen positively in proportion with the network externalities (i.e., the more users there are on the consumer side, the more consumers benefit). The merchants are not affected by the direct network externality on the consumer side, so merchant pricing and welfare remain unchanged. Platform profits are reduced by an amount equal to the direct network externality coefficient, confirming that the reputation mechanism enables the e-commerce platform to transfer part of the surplus value to consumers. The total social welfare level of the e-commerce system increases by an increment of $\frac{1}{2}\alpha_{b}$. Therefore, the symmetry of reputation information contributes toward the total social welfare level and is positively proportional to the network externality. At the same time, we confirm that when the reputation mechanism of the e-commerce platform works, the party that directly benefits is the consumer.

So if there are unfair competitive behaviors, such as “click farming,” “cash rebate for favorable comments,” and “pay per click,” on the merchant side, consumers cannot obtain real information. With this asymmetry of reputation information, what will happen to total social welfare?

### Total social welfare levels when e-commerce product reputation information is asymmetric

When the e-commerce product reputation information is asymmetric, a degree of reputation information asymmetry i ($i=\frac{m_{y}-m_{0}}{m_{0}}$) is introduced to regulate the direct network externality on the consumers’ side.

The utility of an undifferentiated consumer located on platform T is

$$U_{b}^{T}=\theta +\alpha_{b}n_{b}^{T}-\frac{m_{y}-m_{0}}{m_{0}}\alpha_{b}n_{b}^{T}+\beta_{b}{(n}_{s}^{T}+n_{s}^{T,J})-p^{T}-t_{b}n_{b}^{T}.$$

After simplification, we obtain: $U_{b}^{T}=\theta +(1-i)\alpha_{b}n_{b}^{T}+\beta_{b}{(n}_{s}^{T}+n_{s}^{T,J})-p^{T}-t_{b}n_{b}^{T}$

Similarly, the utility of an undifferentiated consumer located on platform J is

$$U_{b}^{J}=\theta +{(1-i)\alpha }_{b}n_{b}^{J}+\beta_{b}(n_{s}^{J}+n_{s}^{T,J})-p^{J}-t_{b}{(1-n}_{b}^{T}).$$ (25)

Since violation behavior, such as brushing orders, requires merchants to pay certain operating costs, if such behavior is investigated and punished, it will increase the penalty cost. To simplify the model, we combine the two costs, which we call the violation cost f. Therefore, the merchant utility is

$$U_{s}^{T}=\theta +\beta_{s}n_{b}^{T}-w^{T}-t_{s}n_{s}^{T}-f$$

$$U_{s}^{J}=\theta +\beta_{s}n_{b}^{J}-w^{J}-t_{s}{(1-n}_{s}^{T}-n_{s}^{T,J})-f$$

$$U_{s}^{T,J}=\theta +\beta_{s}\left(n_{b}^{T}+n_{b}^{J}\right)-w^{T}-w^{J}-t_{s}-2f.$$ (26)

The platform profit formula remains unchanged, and the calculation process is similar to the previous one, and we can obtain consumer pricing, merchant pricing and market share of platform users, the consumer surplus, merchant surplus, platform profit, and total social welfare of the platform system in equilibrium:

$$p^{T^{”}}=p^{J^{”}}={(1-i)\alpha }_{b}-\frac{t_{b}}{2}.$$ (27)

$$w^{T^{”}}=w^{J^{”}}=\frac{\beta_{s}-2f}{3}.$$ (28)

$$n_{b}^{T^{”}}=n_{b}^{J”}=\frac{1}{2},n_{s}^{T^{”}}=1-\frac{\beta_{s}-2f}{6t_{s}},n_{s}^{{T,J}^{"}}=\frac{\beta_{s}-2f}{3t_{s}}-1,{n_{s}^{J}}^{"}=1-\frac{\beta_{s}-2f}{6t_{s}}.$$ (29)

The consumer surplus is

$$CS”=\theta -\frac{1}{2}{(1-i)\alpha }_{b}+\frac{{(\beta }_{s}-2f)\beta_{b}}{6t_{s}}.$$ (30)

The merchant surplus is

$$PS”=\theta -\frac{3}{2}+\frac{2}{3}{(\beta }_{s}-2f)+\frac{2\left(\beta_{s}-2f\right)\left(\theta +\beta_{s}-1\right)-{{(\beta }_{s}-2f)}^{2}-2\theta -\beta_{s}}{6t_{s}}-\frac{{{(\beta }_{s}-2f)}^{2}}{12t_{s}^{2}}.$$ (31)

The profits of the two platforms are:

$$\pi^{T”}+\pi^{J”}=(1-{\begin{array}{c} \left.i)\right. \end{array}\alpha }_{b}-\frac{t_{b}}{2}+\frac{{{(\beta }_{s}-2f)}^{2}}{9t_{s}}.$$ (32)

The total social welfare level of the platform system is

$$W_{3}=CS‘+PS‘+\pi^{T‘}+\pi^{J‘}=2\theta +\frac{1}{2}(1-{i)\alpha }_{b}-\frac{3}{2}-\frac{t_{b}}{2}+(\frac{\beta_{b}}{6t_{s}}+\frac{2}{3})(\beta_{s}-2f)+\frac{6\left(\beta_{s}-2f\right)\left(\theta +\beta_{s}-1\right)-{{(\beta }_{s}-2f)}^{2}-6\theta -3\beta_{s}}{18t_{s}}-\frac{{{(\beta }_{s}-2f)}^{2}}{12t_{s}^{2}}.$$ (33)

### Social welfare effects of reputation information asymmetry

From the above proofs, it can be seen that e-commerce product reputation information asymmetry has certain effects on consumer pricing and surplus, merchant pricing and surplus, e-commerce platform market share and profit, and total social welfare through the asymmetry degree i and the violation penalty cost f, so what is the impact? The theoretical results of total social welfare levels when e-commerce product reputation information is asymmetric and when it is symmetric were compared and analyzed as follows: Suppose β_b_ = 0.20, β_s_ = 0.22, t_b_ = 0.35, t_s_ = 0.30, and θ = 1 [35]. Network externalities were used as moderating variables and took α_b_ = 0.1, 0.2, and 0.3 for sensitivity analysis, respectively.

### Changes in consumer pricing and surplus

From Eqs (27)–(17), (30)–(20), we obtain the following:

Changes in consumer pricing is

$${\Delta p}^{T’}={\Delta p}^{J}’=p^{T^{”}}-p^{T^{'}}=p^{J^{”}}-p^{J^{'}}={{2\alpha }_{b}-i\alpha }_{b}-\frac{{3t}_{b}}{2}+\beta_{s}\frac{3\beta_{b}+\beta_{s}}{4t_{s}}$$ (34)

Changes in consumer surplus is

$$\Delta CS’=CS”-CS’=\frac{3}{8}+(\frac{1}{2}i-2{)\alpha }_{b}-\frac{4f+3{\beta_{s}}^{2}+10\beta_{s}\beta_{b}+3{\beta_{b}}^{2}}{12t_{s}}$$ (35)

**Fig 2:**
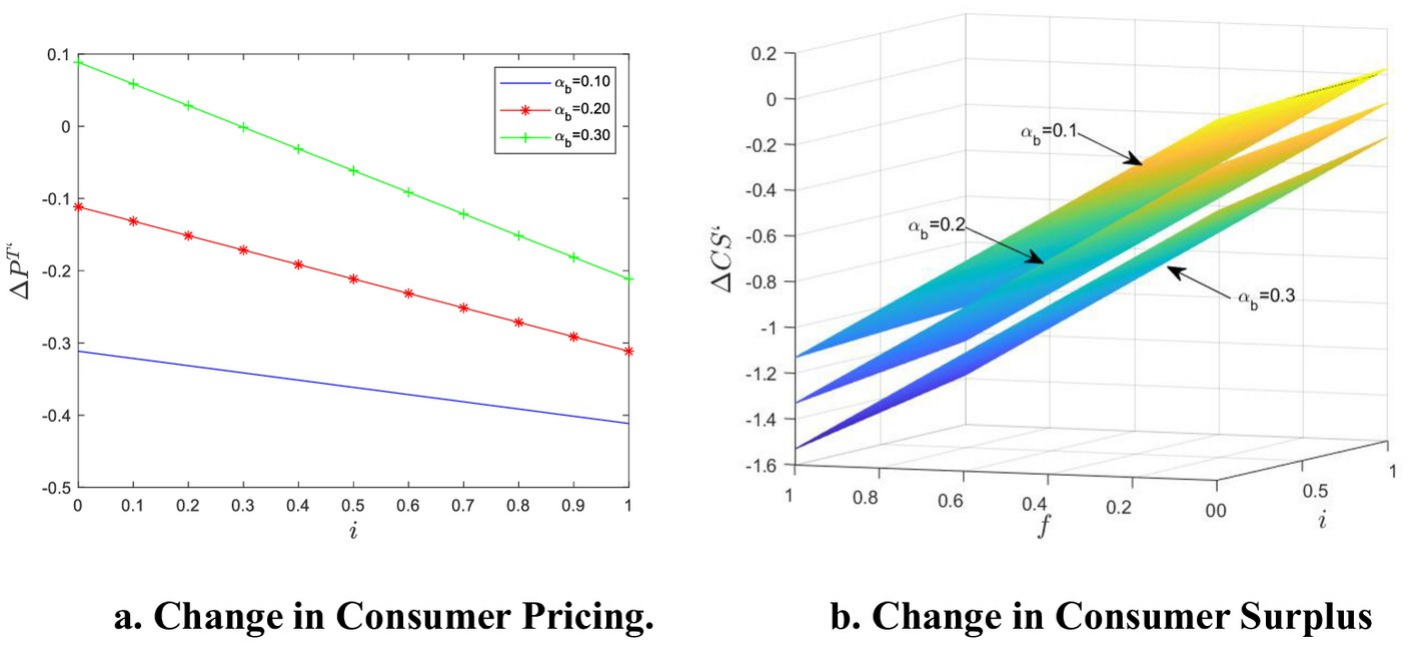
Numerical simulation of consumer pricing and surplus changes when reputation information is asymmetric. a. Change in Consumer Pricing. b. Change in Consumer Surplus.

We used MATLAB software for example analysis and visualization, as shown in Fig 2.

It can be seen that the reputation information asymmetry of e-commerce products has a definite impact on both consumer pricing and surplus. It can be seen from Fig 2a that the change in consumer pricing Δp^T’^ is inversely proportional to i, indicating that the larger the reputation information asymmetry, the lower the platform pricing to consumers, the stronger the direct network externality at the consumer end, and the greater the impact. However, the reduction in consumer pricing does not lead to an increase in welfare, as can be seen from Fig 2b. Only when α_b_ = 0.1 and f < 0.0812 + 0.045i, there is a positive and negative dividing line of ΔCS’, and the reputation information asymmetry increases consumer surplus, otherwise consumer surplus is less than 0. It can be said that reputation information asymmetry has a negative impact on consumers. The change in consumer surplus ΔCS’ is proportional to i and inversely proportional to f and α_b_, indicating that the greater the reputation information asymmetry, the greater the consumer welfare loss, and the higher the cost of violation penalty, and with more platform consumers, the smaller the loss of consumer welfare.

### Changes in merchant pricing and residual

From Eqs (28)–(18) and (31)–(21) we have the following:

Changes in merchant pricing:

$${\Delta w}^{T’}={\Delta w}^{J‘}=w^{T^{”}}-w^{T^{'}}=w^{J^{"}}-w^{J^{'}}=\frac{\beta_{s}-2f}{3}-\frac{\beta_{s}-\beta_{b}}{4}=\frac{\beta_{s}+3\beta_{b}-8f}{12}$$ (36)

Changes in merchant surplus:

$$\Delta PS’=PS”-PS’=\frac{1}{6}\beta_{s}-\frac{1}{2}\beta_{b}-\frac{4}{3}f-\frac{3}{2}+\frac{\left(2\theta -3\right)\beta_{s}+{\beta_{s}}^{2}-\left(4f+2\right)\theta +4f-4f^{2}}{6t_{s}}+\frac{{{{3(4t_{s}-\beta }_{b}-\beta_{s})}^{2}+3\left(\beta_{b}+\beta_{s}-2{t_{s}}^{2}\right)-4(\beta_{s}-2f)}^{2}}{48t_{s}^{2}}$$ (37)

**Fig 3:**
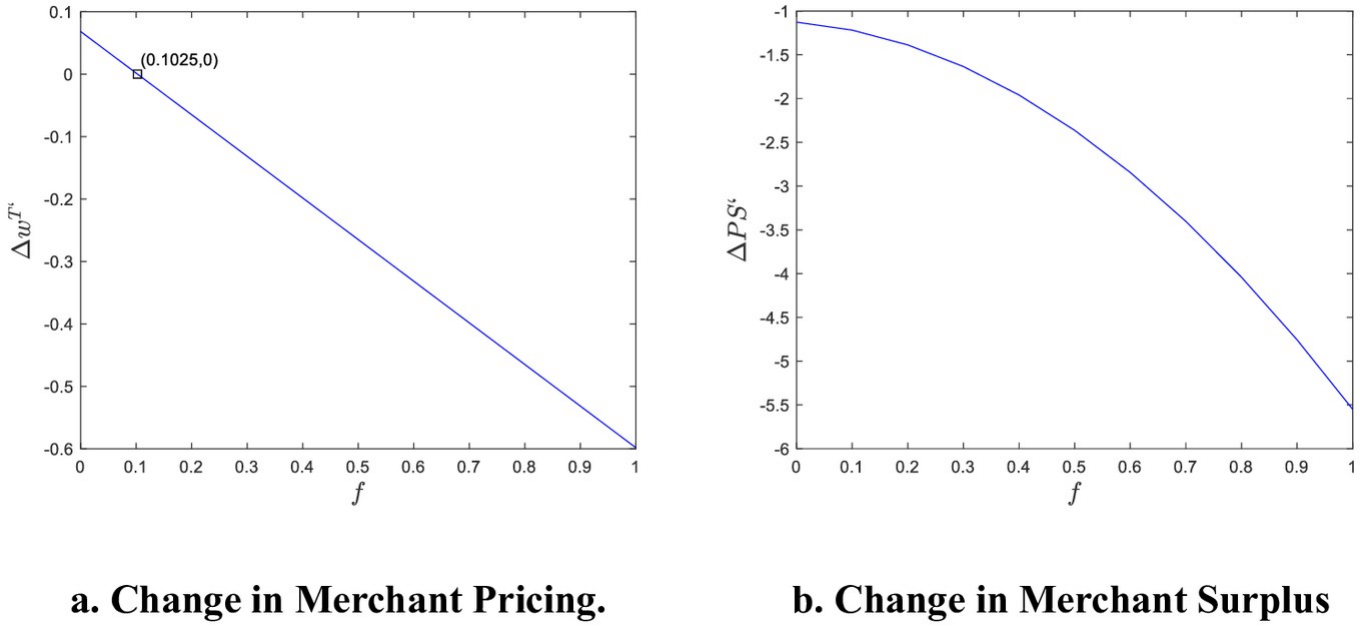
Numerical simulation of merchant pricing and surplus change when reputation information is asymmetric. a. Change in Merchant Pricing. b. Change in Merchant Surplus.

We used MATLAB software for example analysis and visualization, as shown in Fig 3.

It can be seen that e-commerce product reputation information asymmetry has a certain impact on merchant pricing and surpluses, and the main influencing factor is the penalty cost of reputation information violation. As shown in Fig 3a, Δw^T^’ < 0 for f > 0.1025, which means that merchant pricing is reduced after reputation information asymmetry, but merchants do not increase their surplus due to lower pricing, and the change in merchant surplus is less than zero for f > 0 (As can be seen in Fig 3b). Therefore, the reputation information asymmetry reduces the merchant surplus, which may be due to dishonest behavior triggered by a merchant’s low reputation and poor word-of-mouth, thus leading to damaged sales and profit. When f becomes larger, merchants give up illegal business activities such as click farming, and merchant welfare losses decrease.

### Changes in platform market shares and profits

From Eqs (29)–(19) and (32)–(22), we obtain:

Changes in platform market shares:

$$\Delta n_{b}^{T’}=0,\Delta n_{b}^{J’}=0,\Delta n_{s}^{T’}=\Delta n_{s}^{J’}=1-\frac{\beta_{s}-2f}{6t_{s}}-\frac{4t_{s}-\beta_{b}-\beta_{s}}{4t_{s}}=\frac{3\beta_{b}-5\beta_{s}+4f}{12t_{s}},{\Delta n}_{s}^{T,J’}=\frac{\beta_{s}-2f}{3t_{s}}-1-\frac{\beta_{b}+{\beta_{s}-2t}_{s}}{2t_{s}}=\frac{{-\beta }_{s}-3\beta_{b}-4f}{6t_{s}}$$ (38)

Changes in platform profit:

$$\Delta \pi ’=\left(\pi^{T”}+\pi^{J”}\right)-\left(\pi^{T’}+\pi^{J’}\right)={\;2\alpha }_{b}-i\alpha_{b}+-\frac{3}{2}t_{b}+\frac{{8(\beta_{s}-2f)}^{2}+{9({\beta_{s}+\beta }_{b})}^{2}+36\beta_{s}\beta_{b}}{72t_{s}}$$ (39)

**Fig 4:**
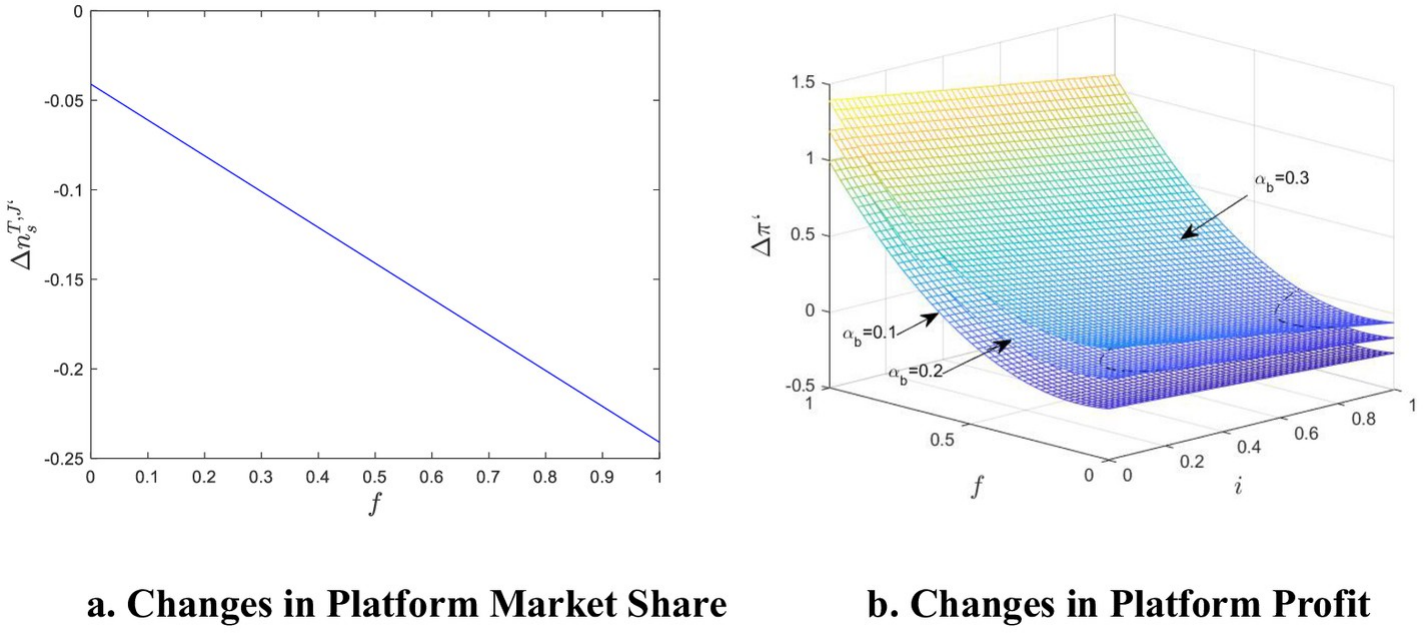
Numerical simulation of changes in platform market share and profit when reputation information is asymmetric. a. Changes in Platform Market Share. b. Changes in Platform Profit.

We used MATLAB software for example analysis and visualization, as shown in Fig 4.

It can be seen that e-commerce product reputation information asymmetry has a certain impact on both platform market share and profit. As Fig 4a shows, e-commerce product reputation information asymmetry increases the number of single-homing merchants on the platform, while the number of multi-homing merchants reduces, and the platform is more willing to focus resources on a platform to create advantages. In addition, the greater the cost of reputation information violation penalty f, the fewer multi-homing merchants there are. Increasing the reputation information violation penalty is conducive to the development of the platform enterprise segmentation industry, avoiding homogeneous competition, and favors the healthy development of the e-commerce platform market. Fig 4b shows that the reputation information asymmetry of e-commerce product does not have a significant impact on the hit on platform profits, Δπ’ is mostly positive, and the higher the cost of reputation information violation penalty, the stronger the direct network externalities, the greater the degree of asymmetry, and the larger the increase in platform profits. It is evident that e-commerce platforms enjoy the benefits of market regulation as well as network externalities but lack the economic impetus to address reputation information asymmetry, which explains the reasons for the persistence of fake reviews.

### Changes in total social welfare of the e-commerce system

From Eqs (33)–(23), we obtain changes in total social welfare:

$$W_{3}\text{-}W_{2}=-\frac{1}{2}\left.i\right.\alpha_{b}-\frac{9}{8}-\frac{{3t}_{b}}{2}-\frac{1}{2}\beta_{s}-\frac{1}{2}\beta_{b}+\left(\frac{\beta_{b}}{6t_{s}}+\frac{2}{3}\right)\left(\beta_{s}-2f\right)+\frac{24\left(\beta_{s}-2f\right)\left(\theta +\beta_{s}-1\right)-{{4(\beta }_{s}-2f)}^{2}-24\theta -12\beta_{s}-9({\beta_{b}+\beta_{s})}^{2}}{72t_{s}}+\frac{{3\left(4t_{s}-\beta_{b}-\beta_{s}\right)}^{2}+3{\left(\beta_{b}+\beta_{s}-2t_{s}\right)}^{2}-4{{(\beta }_{s}-2f)}^{2}}{48t_{s}^{2}}.$$ (40)

**Fig 5:**
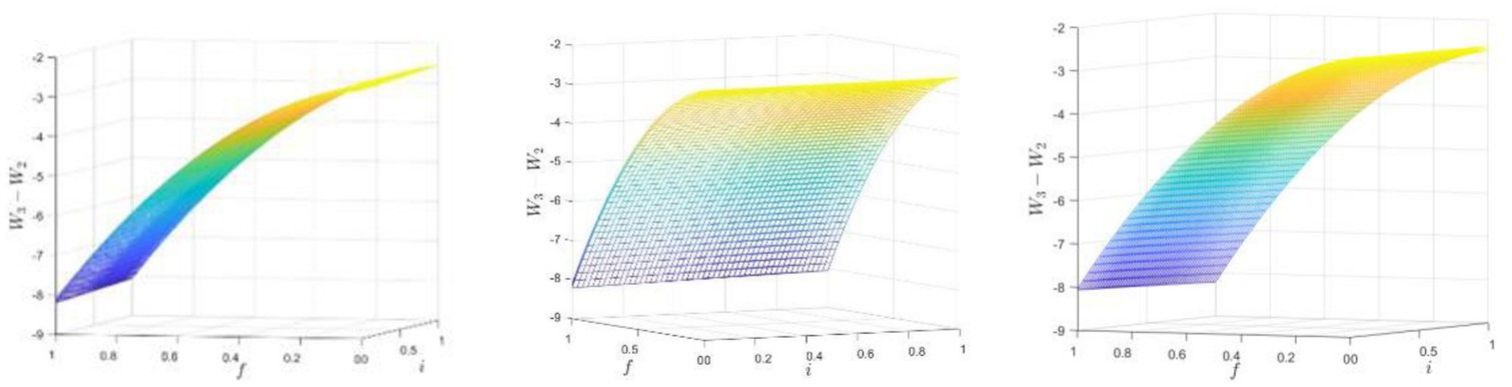
Numerical simulation of the changes in total social welfare when reputation information is asymmetric.

We used MATLAB software for example analysis and visualization, as shown in Fig 5.

From Fig 5, it can be seen that the asymmetry of reputation information of e-commerce products causes a loss of total social welfare, which is mainly affected by the penalty cost of reputation information violation, and has an open-ended downward quadratic function relationship with the penalty cost f. When 0 < f ≤ M, the total social welfare loss increases with the increase of f; when f > M, the total social welfare loss decreases with the increase of f, where $M=\frac{3t_{s}(-\beta_{b}-2\beta_{s}+2)-16t_{s}^{2}-8\theta t_{s}+\beta_{s}}{6-3\beta_{s}+4t_{s}-2\beta_{s}t_{s}}$. For 0 < f ≤1, the total social welfare loss is inversely proportional to f. In addition, the sensitivity analysis for α_b_ is taken as 0.1, 0.2, and 0.3, respectively, and the three surfaces are close to overlap, but when the direct network externality is very large, the total social welfare loss will be smaller. The degree of asymmetry adjusts the speed of the change in social welfare loss. When i becomes larger, social welfare loss accelerates.

## Conclusion and management insights

In this study, we found that reputation mechanisms can effectively increase consumer surplus and total social welfare, and this increases as the size of the online consumer user base increases.

When the reputation information of e-commerce products is asymmetric, the consumer surplus is reduced, which is proportional to the degree of reputation information asymmetry and inversely proportional to the penalty cost of violation and the direct network externalities of consumers. The merchant’s surplus decreases, and the merchant’s welfare loss decreases with the increase in the penalty cost of reputation information violation. The platform revenue increases and is proportional to the network externality, the penalty cost of violation, and the degree of reputation information asymmetry. The platform lacks the economic motivation to strictly control reputation information asymmetry. Total social welfare is reduced, which is inversely proportional to the cost of punishing violations and the direct network externalities. Increasing the cost of violation punishment, increasing the scale of consumers, and reducing the number of fake reviews can reduce the loss of social welfare.

It is not difficult to see that information asymmetry has a negative impact on consumers, merchants, and the total welfare of society but promotes a high probability of profitability for e-commerce platforms, which is extremely unfavorable for the development of the e-commerce market. Therefore, we propose the following:

(1) Establish a management mechanism for joint supervision by e-commerce platforms, third-party agencies, and social organizations. E-commerce platforms should take up social responsibility to improve the credit evaluation mechanisms of merchants, reduce the weight of sales ranking and consumer reviews in reputation mechanism, integrate business registration, tax payment status, integrity records, and other information into the merchant credit evaluation system, and fulfill supervision and management obligations. Establish a third-party e-commerce product reputation information supervision platform, integrate effective information of merchants collected by each platform, achieve information sharing while obtaining more accurate comprehensive evaluations, improve the level of false comment identification and supervision through self-machine learning, AI, and other technologies, and assist e-commerce platform supervision to improve supervision efficiency [40]. Organizations such as the FTC and market supervision and management should strengthen the enforcement of various laws and regulations, such as the Anti-Unfair Competition Law, the Electronic Commerce Law, and the Consumer Protection Law, and implement the blacklist system for illegal businesses to improve the effectiveness of social supervision.
(2) Severely punish click farming, increase the cost of penalties for violating reputation information, set legal standards, and impose severe penalties on companies that disrupt the order of competitive order of the marketplace. In 2024, the FTC improved the maximum civil penalty for false reviews to $51,744 per violation (https://www.163.com). In addition, it is necessary to strictly enforce the primary responsibility of platforms and limit the click farming behavior of merchants by strictly specifying punitive measures in the registration rules. Platforms that fail to fulfill their regulatory responsibilities should also be punished in accordance with the law. If they are suspected of committing a crime, they should be handed over to the public security department for criminal responsibility in accordance with the law, which will enhance the deterrent effect of the country’s rule of law.
(3) Make full use of the externality of the platform network effect to enhance the competitiveness of enterprises. From this study’s conclusion, it can be seen that network externality can increase consumer surplus, platform profit, and total social welfare. The effect of direct network externality on the consumer side is determined by the number of consumers, the number of people who actively participating in the online review, and the objective authenticity of the reviews. Platform enterprises can increase the number of users through marketing activities, social media promotion and other forms, personalized recommendations through data analysis and mining, optimize user experience, and enhance network externalities. As one of the main parties of value co-creation in the e-commerce market, platform enterprises can provide appropriate incentives to customers with honest reviews, conduct investigations, corrections, and punish merchants who delete negative comments. Only by eliminating unfair competition at source can we better establish the reputation and image of the platform, attract more users, and gain a long-term competitive advantage for the company.

This study examined the effectiveness of the reputation mechanism in the e-commerce platform market and conducted a game argumentation on the changes in consumers, merchants, platforms, and total social welfare under asymmetric reputation information. The conclusions obtained expand the theory related to the mechanism of the impact of asymmetry of reputation information and lay the theoretical foundations for future empirical research. In future research, we can start with the following aspects: (1) This study considers the market situation of duopolistic e-commerce platforms, consumers’ single-homing and linear costs and benefits, but with the increasingly fierce competition, multiple platforms are emerging and the fact that consumers are mostly assigned to multiple platforms is closer to real life. An improved general competition model can be considered to further verify the robustness of the conclusion. (2) This study is based on theoretical arguments and verified by example analysis. There are no actual data. The follow-up study can consider adding actual data from an e-commerce platform for verification. (3) This study only considered only two types of reputation information: feedback evaluation and sales ranking. Subsequent research can complement, expand, and optimize the design of the content of the reputation mechanism to make it work better.

## Supporting information

S1 File (DOCX)
