# Supplementary material for: The social welfare effect of e-commerce product reputation information asymmetry from the perspective of network externality
Source: PLoS One. 2025 Jan 2;20(1):e0313852. doi: 10.1371/journal.pone.0313852 (PMC11694973; doi:10.1371/journal.pone.0313852)
Supplement: S1 File — (DOCX) [file pone.0313852.s001.docx]

1.There is no data set, all the data is in the manuscript.

2.The fourth part of the article which is Social welfare effects of reputation information asymmetry，these following data are used for example analysis**.**

**Example Analysis**

Suppose β_b_ = 0.20, β_s_ = 0.22, t_b_ = 0.35, t_s_ = 0.30, and θ = 1. Network externalities were used as moderating variables and took α_b_ = 0.1, 0.2, and 0.3 for sensitivity analysis, respectively.

We used MATLAB 2021b software for example analysis and visualization, as shown in Fig 2-5.

**Figure 2a Code is as follows:**

clear;clc;

α_b_ = 0.10;

β_b_ = 0.20;

β_s_ = 0.22;

t_b_ = 0.35;

t_s_ = 0.30;

θ = 1;

i = 0:0.1:1;

p = i*ab + ab -3*tb/2 + bs*((3*bb+bs)/4*ts);

plot(i,p,'b-','DisplayName','\alpha_b=0.10');

hold on;

α_b_ = 0.20;

p = i*ab + ab -3*tb/2 + bs*((3*bb+bs)/4*ts);

plot(i,p,'r-*','DisplayName','\alpha_b=0.20');

hold on;

α_b_ = 0.30;

p = i*ab + ab -3*tb/2 + bs*((3*bb+bs)/4*ts);

plot(i,p,'g-+','DisplayName','\alpha_b=0.30');

hold on;

legend;

L(1) = xlabel('$ i $','interpreter','latex','FontSize',15);

L(2) = ylabel('$ \Delta P^{T`} $','interpreter','latex','FontSize',15);

**Figure 2b Code is as follows:**

clear;clc;

α_b_ = 0.10;

β_b_ = 0.20;

β_s_ = 0.22;

t_b_ = 0.35;

t_s_ = 0.30;

θ = 1;

i = linspace(0,1,50);

f = linspace(0,1,50);

[I,F] = meshgrid(i,f);

CS = 3/8 - (1/2)*I*ab -(3/2)*ab - (4*F+3*bs*bs+10*bs*bb+3*bb*bb)/(12*ts);

f2 = linspace(0,1,50);

i2=((3/8 -(3/2)* ab - (4*f2+3*bs*bs+10*bs*bb+3*bb*bb)/(12*ts))*2)/ab;

cs2 = i2-i2;

mesh(I,F,CS);

hold on;

plot3(i2,f2,cs2,'k-');

hold on;

α_b_ = 0.2;

CS = 3/8 - (1/2)*I*ab -(3/2)*ab - (4*F+3*bs*bs+10*bs*bb+3*bb*bb)/(12*ts);

i2=((3/8 -(3/2)* ab - (4*f2+3*bs*bs+10*bs*bb+3*bb*bb)/(12*ts))*2)/ab;

cs2 = i2-i2;

hold on;

mesh(I,F,CS);

hold on;

plot3(i2,f2,cs2,'g+');

hold on;

α_b_ = 0.3;

CS = 3/8 - (1/2)*I*ab -(3/2)*ab - (4*F+3*bs*bs+10*bs*bb+3*bb*bb)/(12*ts);

i2=((3/8 -(3/2)* ab - (4*f2+3*bs*bs+10*bs*bb+3*bb*bb)/(12*ts))*2)/ab;

cs2 = i2-i2;

mesh(I,F,CS);

hold on;

plot3(i2,f2,cs2,'r*');

axis([0,1,0,1]);

L(1) = xlabel('$i$','interpreter','latex','FontSize',15);

L(2) = ylabel('$f$','interpreter','latex','FontSize',15);

L(3) = zlabel('$\Delta CS`$','interpreter','latex','FontSize',15);


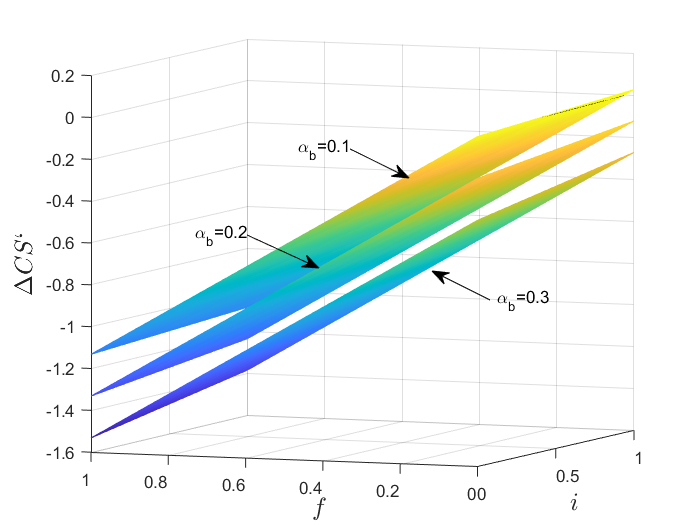


a. Change of consumer pricing b. Change of consumer surplus

Figure 2. Numerical simulation of consumer pricing and surplus changes when reputation information is asymmetric

**Figure 3a Code is as follows:**

clear;clc;

α_b_ = 0.10;

β_b_ = 0.20;

β_s_ = 0.22;

t_b_ = 0.35;

t_s_ = 0.30;

θ = 1;

f= 0:0.1:1;

w = (bs+3*bb-8*f)/12;

plot(f,w,'b-');

hold on;

w= 0;

f=(w*12-bs-3*bb)/(-8);

plot(f,w,'ks');

text(0.108,0.024,'(0.1025,0)');

L(1) = xlabel('$ f $','interpreter','latex','FontSize',15);

L(2) = ylabel('$ \Delta w^{T`} $','interpreter','latex','FontSize',15);

**Figure 3b Code is as follows:**

clear;clc;

α_b_ = 0.10;

β_b_ = 0.20;

β_s_ = 0.22;

t_b_ = 0.35;

t_s_ = 0.30;

θ = 1;

f= 0:0.1:1;

ps = (1/6)*bs-(1/2)*bb-(4/3)*f-3/2 + ((2*cta-3)*bs+bs*bs-(4*f+2)*cta+4*f-4*f.*f)/6*ts ...

+ (3*(4*ts-bb-bs)*(4*ts-bb-bs)+3*(bb+bs-2*ts*ts)-4*(bs-2*f).*(bs-2*f))/(48*ts*ts);

plot(f,ps,'b-');

hold on;

L(1) = xlabel('$ f $','interpreter','latex','FontSize',15);

L(2) = ylabel('$ \Delta PS` $','interpreter','latex','FontSize',15);

a. Change of merchant pricing b. Change of merchant surplus

Figure 3. Numerical simulation of merchant pricing and surplus change when reputation information is asymmetric

**Figure 4a Code is as follows:**

clear;clc;

α_b_ = 0.10;

β_b_ = 0.20;

β_s_ = 0.22;

t_b_ = 0.35;

t_s_ = 0.30;

θ = 1;

f= 0:0.1:1;

n = (-bs-3*bb-4*f)/6*ts;

plot(f,n,'b-');

hold on;

L(1) = xlabel('$ f $','interpreter','latex','FontSize',15);

L(2) = ylabel('$ \Delta n^{T,J`}_s $','interpreter','latex','FontSize',15);

**Figure 4b Code is as follows:**

clear;clc;

α_b_ = 0.10;

β_b_ = 0.20;

β_s_ = 0.22;

t_b_ = 0.35;

t_s_ = 0.30;

θ = 1;

i = linspace(0,1,50);

f = linspace(0,1,50);

[I,F] = meshgrid(i,f);

PAI =I*ab + ab - (3/2)*tb + (8*(bs-2*F).*(bs-2*F)+9*(bs+bb)^2+36*bs*bb)/(72*ts);

f2 = linspace(0,1,50);

i2=-(ab - (3/2)*tb + (8*(bs-2*f2).*(bs-2*f2)+9*(bs+bb)^2+36*bs*bb)/(72*ts))/ab;

pai2 = i2-i2;

Fig = mesh(I,F,PAI);

hold on;

plot3(i2,f2,pai2,'k--');

hold on;

α_b_ = 0.2;

PAI =I*ab + ab - (3/2)*tb + (8*(bs-2*F).*(bs-2*F)+9*(bs+bb)^2+36*bs*bb)/(72*ts);

i2=-(ab - (3/2)*tb + (8*(bs-2*f2).*(bs-2*f2)+9*(bs+bb)^2+36*bs*bb)/(72*ts))/ab;

pai2 = i2-i2;

Fig = mesh(I,F,PAI);

hold on;

plot3(i2,f2,pai2,'k--');

hold on;

α_b_ = 0.3;

PAI =I*ab + ab - (3/2)*tb + (8*(bs-2*F).*(bs-2*F)+9*(bs+bb)^2+36*bs*bb)/(72*ts);

i2=-(ab - (3/2)*tb + (8*(bs-2*f2).*(bs-2*f2)+9*(bs+bb)^2+36*bs*bb)/(72*ts))/ab;

pai2 = i2-i2;

Fig = mesh(I,F,PAI);

hold on;

plot3(i2,f2,pai2,'k--');

hold on;

axis([0,1,0,1]);

L(1) = xlabel('$i$','interpreter','latex','FontSize',15);

L(2) = ylabel('$f$','interpreter','latex','FontSize',15);

L(3) = zlabel('$\Delta \pi`$','interpreter','latex','FontSize',15);


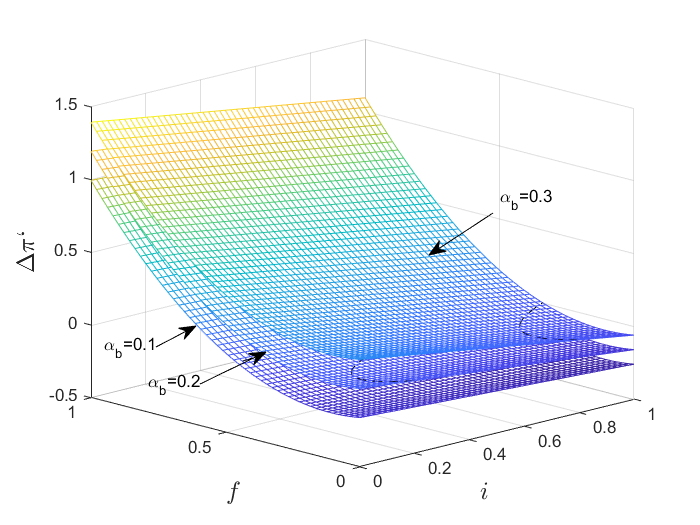


a. platform market share change b. platform profit change

Figure 4. Numerical simulation of platform market share and profit change when reputation information is asymmetric

**Figure 5 Code is as follows:**

clear;clc;

α_b_ = 0.10;

β_b_ = 0.20;

β_s_ = 0.22;

t_b_ = 0.35;

t_s_ = 0.30;

θ = 1;

i = linspace(0,1,50);

f = linspace(0,1,50);

[I,F] = meshgrid(i,f);

W = (1/2)*I*ab - (1/2)*ab - 9/8 - (3*tb)/2 - (1/2)*bs -(1/2)*bb+ (bb/(6*ts)+2/3)*(bs-2*F)...

+ (24*(bs-2*F)*(cta+bs-1)-4*(bs-2*F).*(bs-2*F)-24*cta-12*bs-9*(bb+bs)*(bb+bs))/(72*ts)...

+ (3*(4*ts-bb-bs)*(4*ts-bb-bs)+3*(bb+bs-2*ts)*(bb+bs-2*ts)-4*(bs-2*F).*(bs-2*F))/(48*ts*ts);

Fig = mesh(I,F,W);

hold on;

α_b_ = 0.2;

W = (1/2)*I*ab - (1/2)*ab - 9/8 - (3*tb)/2 - (1/2)*bs -(1/2)*bb+ (bb/(6*ts)+2/3)*(bs-2*F)...

+ (24*(bs-2*F)*(cta+bs-1)-4*(bs-2*F).*(bs-2*F)-24*cta-12*bs-9*(bb+bs)*(bb+bs))/(72*ts)...

+ (3*(4*ts-bb-bs)*(4*ts-bb-bs)+3*(bb+bs-2*ts)*(bb+bs-2*ts)-4*(bs-2*F).*(bs-2*F))/(48*ts*ts);

Fig = mesh(I,F,W);

hold on;

α_b_ = 0.3;

W = (1/2)*I*ab - (1/2)*ab - 9/8 - (3*tb)/2 - (1/2)*bs -(1/2)*bb+ (bb/(6*ts)+2/3)*(bs-2*F)...

+ (24*(bs-2*F)*(cta+bs-1)-4*(bs-2*F).*(bs-2*F)-24*cta-12*bs-9*(bb+bs)*(bb+bs))/(72*ts)...

+ (3*(4*ts-bb-bs)*(4*ts-bb-bs)+3*(bb+bs-2*ts)*(bb+bs-2*ts)-4*(bs-2*F).*(bs-2*F))/(48*ts*ts);

Fig = mesh(I,F,W);

hold on;

axis([0,1,0,1]);

L(1) = xlabel('$i$','interpreter','latex','FontSize',15);

L(2) = ylabel('$f$','interpreter','latex','FontSize',15);

L(3) = zlabel('$W_3-W_2$','interpreter','latex','FontSize',15);


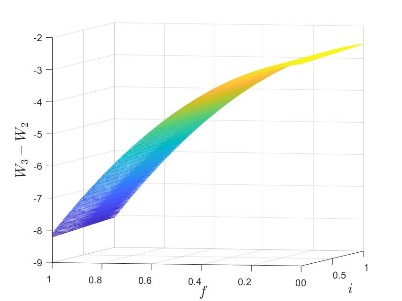

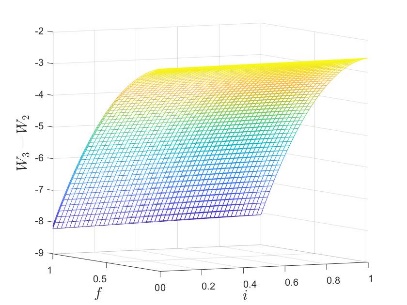

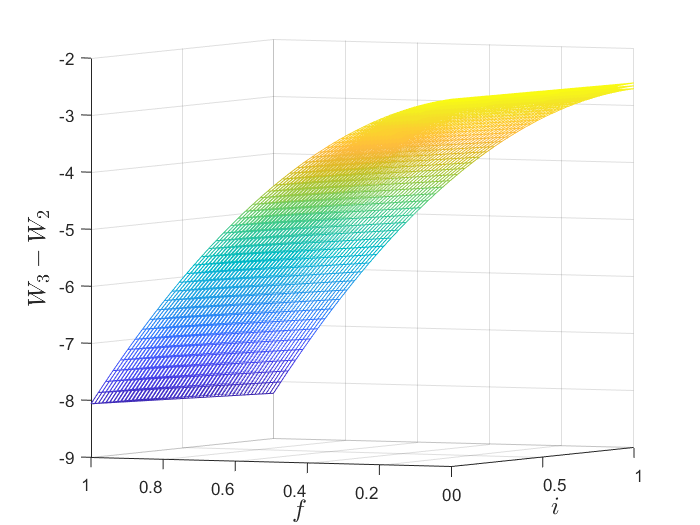


Figure 5. Numerical simulation of the change in total social welfare when reputation information is asymmetric
